# Fatty acids suppress the steroidogenesis of the MA-10 mouse Leydig cell line by downregulating CYP11A1 and inhibiting late-stage autophagy

**DOI:** 10.1038/s41598-021-92008-2

**Published:** 2021-06-15

**Authors:** Chien Huang, Hsiu-Ju Hsu, Mu-En Wang, Meng-Chieh Hsu, Leang-Shin Wu, De-Shien Jong, Yi-Fan Jiang, Chih-Hsien Chiu

**Affiliations:** 1 Laboratory of Animal Physiology, Department of Animal Science and Technology, National Taiwan University, Taipei, 10617 Taiwan; 2 Department of Pathology, Duke University School of Medicine, Durham, NC USA; 3 Biochemistry Section, Surgical Neurology Branch, National Institute of Neurological Disorders and Stroke, National Institutes of Health, Bethesda, MD USA; 4 Graduate Institute of Molecular and Comparative Pathobiology, School of Veterinary Medicine, National Taiwan University, Taipei, 10617 Taiwan

**Keywords:** Cell biology, Endocrinology

## Abstract

Obese men have lower circulating testosterone than men with an optimal body mass index. Elevated fatty acids (FAs) caused by obesity have been reported to suppress the steroidogenesis of Leydig cells. Recent studies have demonstrated that autophagy regulates steroidogenesis in endocrine cells; however, few studies have investigated the molecular mechanisms of FA-impaired steroidogenesis. To study FA regulation in the steroidogenesis of Leydig cells, MA-10 cells were treated with an FA mixture and co-treated with 8-Br-cAMP to stimulate the steroidogenesis capacity. We showed that FAs led to cellular lipid accumulation and decreased steroidogenesis of MA-10 cells, and FA-suppressed steroidogenesis was largely recovered by P5 treatment but not by 22R-OHC treatment, suggesting the primary defect was the deficiency of CYP11A1. To examine the involvement of autophagy in the steroidogenesis of Leydig cells, we treated MA-10 cells with autophagy regulators, including rapamycin, bafilomycin, and chloroquine. Inhibition of late-stage autophagy including FA-upregulated Rubicon suppressed the steroidogenesis of MA-10 cells. More interestingly, Rubicon played a novel regulatory role in the steroidogenesis of MA-10 cells, independent of inhibitors of late-stage autophagy. Collectively, this study provides novel targets to investigate the interaction between FAs and steroidogenesis in steroidogenic cells.

## Introduction

Clinical investigations have shown that obese men are at higher risks of infertility than those with a normal body mass index (BMI), likely because of their lower sperm counts and worse semen quality^1–3^. Additionally, the serum testosterone levels are significantly lower in obese men but can be recovered by gastric bypass surgery^4^. Obesity is usually accompanied by hyperlipidemia, comprising increased free fatty acids (FFAs) and triglycerides (TGs). Previous studies have shown that both FFAs and TGs can impair steroidogenesis in primary mouse Leydig cells and mouse Leydig cell lines^5,6^. According to Rone et al., steroid biosynthesis in the mouse Leydig cell line MA-10 increased after blocking the import of FFAs into the mitochondria^7^. Decreased testosterone production by the Leydig cell line caused by palmitic acid-induced apoptosis was restored by inhibiting endoplasmic reticulum stress^6^. However, although previous studies have identified FFA inhibition in the steroid production of Leydig cells, few have investigated the molecular mechanisms underlying the suppressed steroidogenesis of Leydig cells under physiological conditions of FFAs during obesity.

Recent studies have found that autophagy regulates hormone secretion by trafficking stored steroid precursor^8^, increasing the degradation of (pro)insulin^9^ and other factors^10^. Autophagy is a critical process responsible for providing sufficient nutrients, performing self-renewal, and protecting cells from cellular stress^11^. In response to different nutrient conditions, the activation of early-stage autophagy is tightly regulated by AMPK-mTOR-Ulk1 signaling^12^. Next, during maturation, the newly synthesized phagophores engulf selective components with the help of mature LC3 and cargo protein p62^13,14^. After maturation, autophagosomes fuse with lysosomes for further clearance^15^. Although fatty acids (FAs) impair autophagy in many cell types, such as hepatocytes, kidney proximal tubular cells, and endothelial cells^16–18^, the mechanism by which FAs affect autophagy in steroidogenic cells remains unclear. Previous studies have demonstrated that FAs inhibit AMPK-mTOR-Ulk1 signaling, which is important for early-stage autophagy in hepatocytes^19,20^. Other studies have shown that FAs interfere with late-stage autophagy by inhibiting fusion between autophagosomes and lysosomes^21–23^ and inactivating lysosomal acidification^24^.

Although previous studies have shown that FAs decrease the steroidogenesis of Leydig cells and autophagy regulates steroid production, no study has investigated the connection between FA-suppressed steroidogenesis and autophagy. Therefore, we hypothesized that FAs suppressed steroid production via disturbing autophagy in Leydig cells. In this study, we first constructed a suppressed steroidogenesis model using the mouse Leydig cell line MA-10 treated with a suitable concentration and composition of FAs. To test our hypothesis, we also applied autophagy regulators to FA-treated MA-10 cells to further investigate the underlying molecular mechanisms. Accordingly, we examined whether the regulation of autophagy homeostasis improved FA-suppressed steroidogenic signaling.

## Results

### Fatty acids induce lipid accumulation and inhibit the steroidogenesis of MA-10 cells

**Figure 1:**
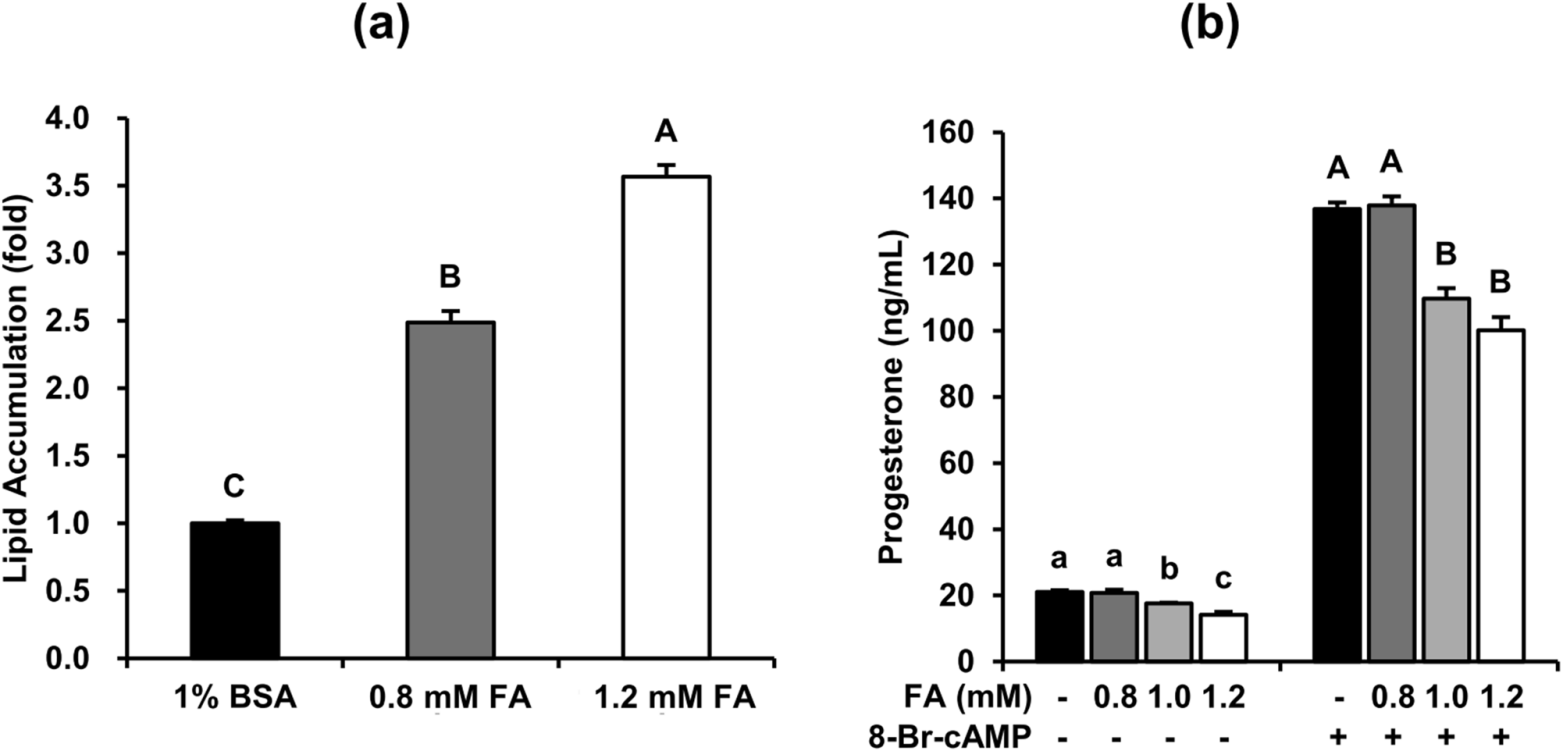
Fatty acids induce lipid accumulation and inhibit the steroidogenesis of MA-10 cells. MA-10 cells were treated with 1% BSA, 0.8 mM (0.4 mM PA mixed with 0.4 mM OA) to 1.2 mM fatty acids (0.6 mM PA mixed with 0.6 mM OA) for 48 h. The cells were then stained with Nile Red to quantify lipid accumulation and with Hoechst 33,342 to normalize the cell numbers. (**a**) The relative lipid accumulation under different FA dosages is shown as the means ± standard errors of the mean (SEM) (n = 3). After FA treatment for 48 h, MA-10 cells were treated with or without 50 µM 8-Br-cAMP for 4 additional hours to induce steroidogenesis. Conditioned medium was then collected to measure steroidogenesis. (**b**) The progesterone levels in the conditioned medium are shown as means ± SEM (n = 3). Different letters represent a significant difference between groups analyzed by one-way ANOVA followed with Duncan’s multiple comparisons (p < 0.05).

To understand whether increasing the FA levels would inhibit the steroidogenesis of Leydig cells, we first constructed an in vitro platform using mouse Leydig cell line MA-10 treated with different concentrations of an FA mixture for 48 h. As shown in Fig. 1a, both 0.8-mM and 1.2-mM of FA treatment significantly increased lipid accumulation in MA-10 cells after 48-h induction. Furthermore, to investigate the capacity of steroidogenesis under FA challenge, MA-10 cells were treated with FA for 48 h and then treated with 50 μM 8-Br-cAMP for 4 h to stimulate steroidogenesis. Additionally, progesterone (P4) secretion in both basal and 8-Br-cAMP-stimulated states was significantly decreased by both 1.0-mM and 1.2-mM FA treatment but was not significantly decreased in both states under 0.8-mM FA treatment (Fig. 1b). Given these data, we applied 1.2 mM FA for 48 h and 50 μM 8-Br-cAMP for 4 h to MA-10 cells as an in vitro model of basal and stimulated steroidogenesis of Leydig cells in further experiments.

### Fatty acid-suppressed steroidogenesis by impairing steroidogenic signaling is largely recovered by P5 treatment in MA-10 cells

**Figure 2:**
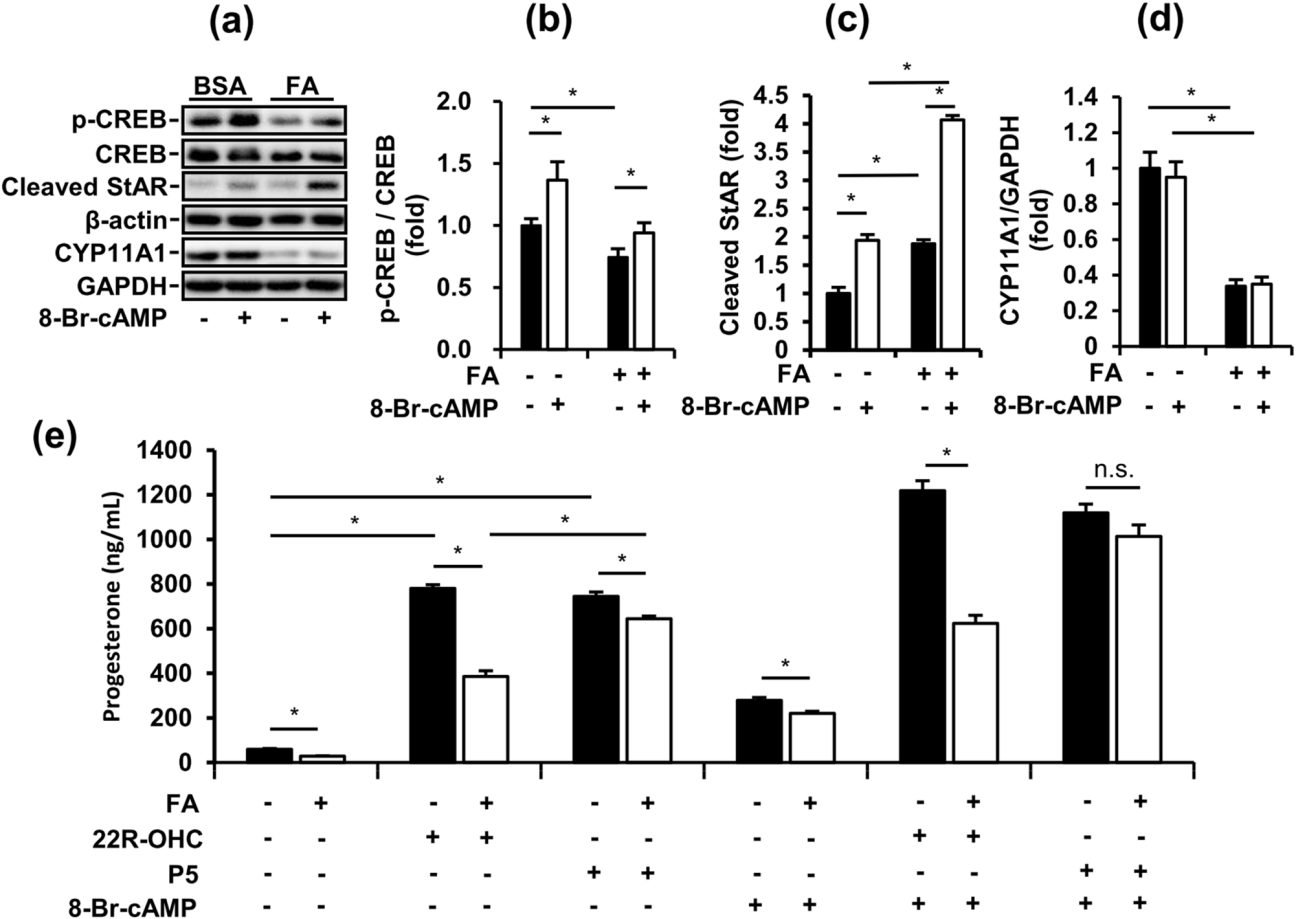
Fatty acid-suppressed steroidogenesis through impaired steroidogenic signaling is largely recovered by P5 treatment in MA-10 cells. (**a**) Representative blots in FA-induced MA-10 cells after 4-h 8-Br-cAMP treatment are shown. (**b**), (**c**), (**d**) Quantifications of blots normalized by β-actin or GAPDH are shown as means ± SEM (n = 3). (**e**) The progesterone levels from FA-induced MA-10 cells treated with or without 50 µM 8-Br-cAMP, 1 µg/mL of P5 and 10 µg/mL of 22R-OHC for 4 additional hours. The data are represented as means ± SEM (n = 3). *P < 0.05. n.s.: no significant difference.

To examine the effects of FA exposure on the steroidogenesis of Leydig cells, we analyzed steroidogenic signaling in FA-induced MA-10 cells using western blotting. The phosphorylation of cAMP response element-binding protein (CREB) and expression of CYP11A1 in MA-10 cells were significantly decreased by FA treatment for 48 h (Fig. 2a, b, d). CREB is a crucial transcription factor in steroidogenesis, and CYP11A1 is involved in the conversion of cholesterol to pregnenolone. Conversely, cleaved StAR, which transports cholesterol into mitochondria, was significantly upregulated in MA-10 cells by FA treatment for 48 h in both the basal and stimulated states (Fig. 2c). Interestingly, 8-Br-cAMP stimulation significantly increased the phosphorylation of CREB and expression of cleaved StAR but had no effects on the expression of CYP11A1 in the BSA and FA groups. Therefore, we hypothesized that FA-suppressed P4 secretion from MA-10 cells was dependent on CYP11A1 downregulation. To test our hypothesis, we applied the steroidogenic substrates 22R-OHC and P5 to our MA-10 model to evaluate specific steps of steroidogenesis. In the basal state, P4 secretion from MA-10 cells was significantly increased by both 22R-OHC and P5 treatment but was also decreased by FAs (Fig. 2e). Additionally, in the 8-Br-cAMP-stimulated state, P4 secretion from MA-10 cells was further increased by 22R-OHC and P5 treatment. More importantly, in the 8-Br-cAMP-stimulated state, FA-suppressed P4 secretion from MA-10 cells was totally restored by P5 but not by 22R-OHC treatment. However, in the basal state, FA-suppressed P4 secretion from MA-10 cells was only partially recovered by P5 treatment. Taken together, by providing a P5 substrate to undergo CYP11A1 catalysis, the steroidogenesis of MA-10 could occur normally even under FA challenge.

### Fatty acids disrupt autophagy and increase ER stress in MA-10 cells

**Figure 3:**
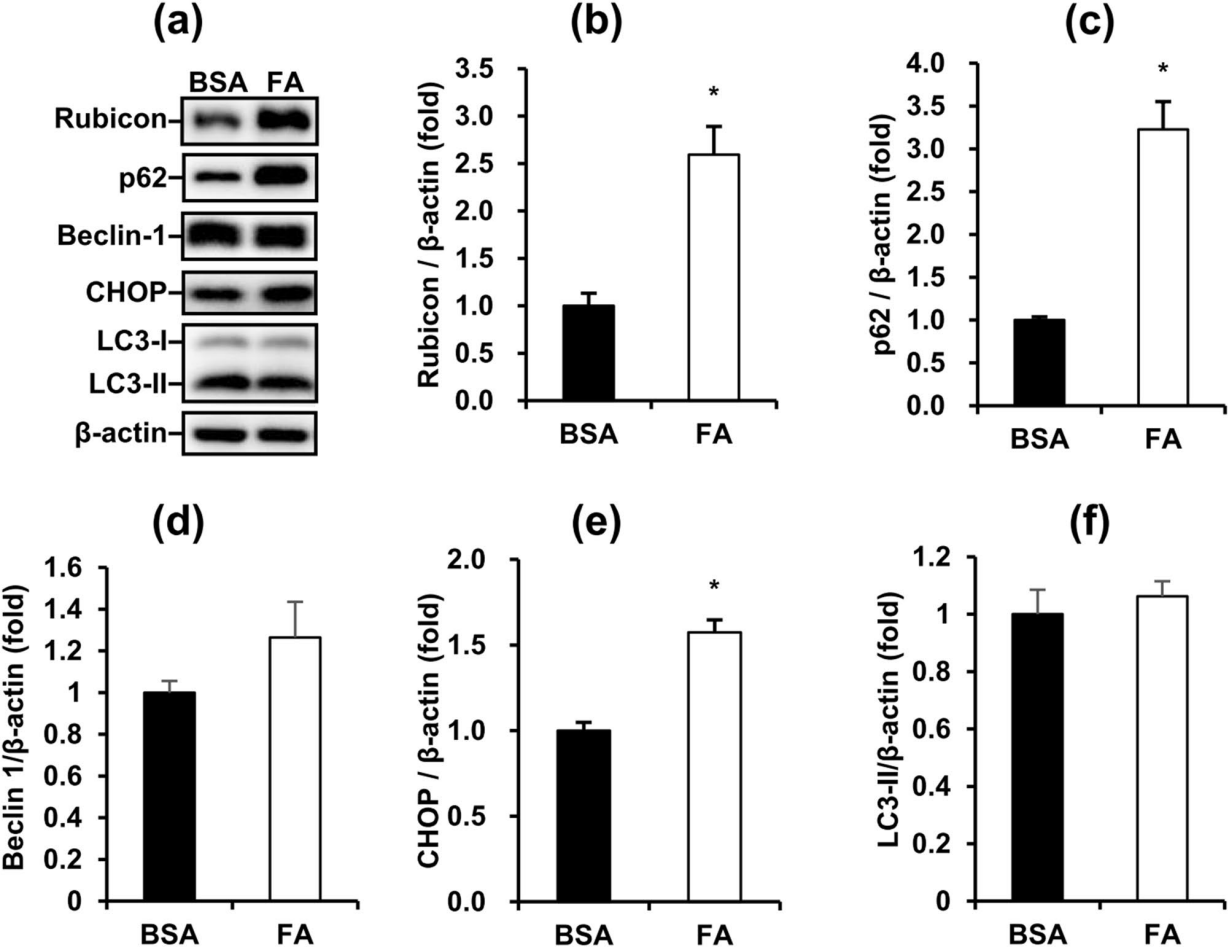
Fatty acids disrupt autophagy and increase ER stress in MA-10 cells. (**a**) Representative blots of autophagy and ER stress markers in FA-induced MA-10 cells are shown. (**b**), (**c**), (**d**), (**e**), (**f**) Quantification of blots normalized by β-actin are shown as means ± SEM (n = 3). *P < 0.05.

Because many studies had shown that cellular lipid content alters autophagic function^25,26^, we speculated whether FA suppressed the steroidogenesis of Leydig cells by regulating autophagy. Therefore, we analyzed the major markers involved in the autophagy process. The late-stage blocker of autophagy Rubicon and cargo protein p62 were significantly upregulated in MA-10 cells by FA treatment for 48 h (Fig. 3a, b, c). This pattern indicated that FAs inhibited the late-stage autophagy in MA-10 cells. Furthermore, the ER stress marker CHOP was upregulated in MA-10 cells by FA treatment for 48 h (Fig. 3e). However, the core unit of the initiation complex Beclin-1 and autophagosome marker LC3-II in MA-10 cells were not changed by FA treatment for 48 h (Fig. 3d,f). In summary, we demonstrated that FA inhibited the late-stage of autophagy and increased ER stress in MA-10 cells.

### Fatty acids simultaneously inhibit and induce early-stage autophagy in MA-10 cells

**Figure 4:**
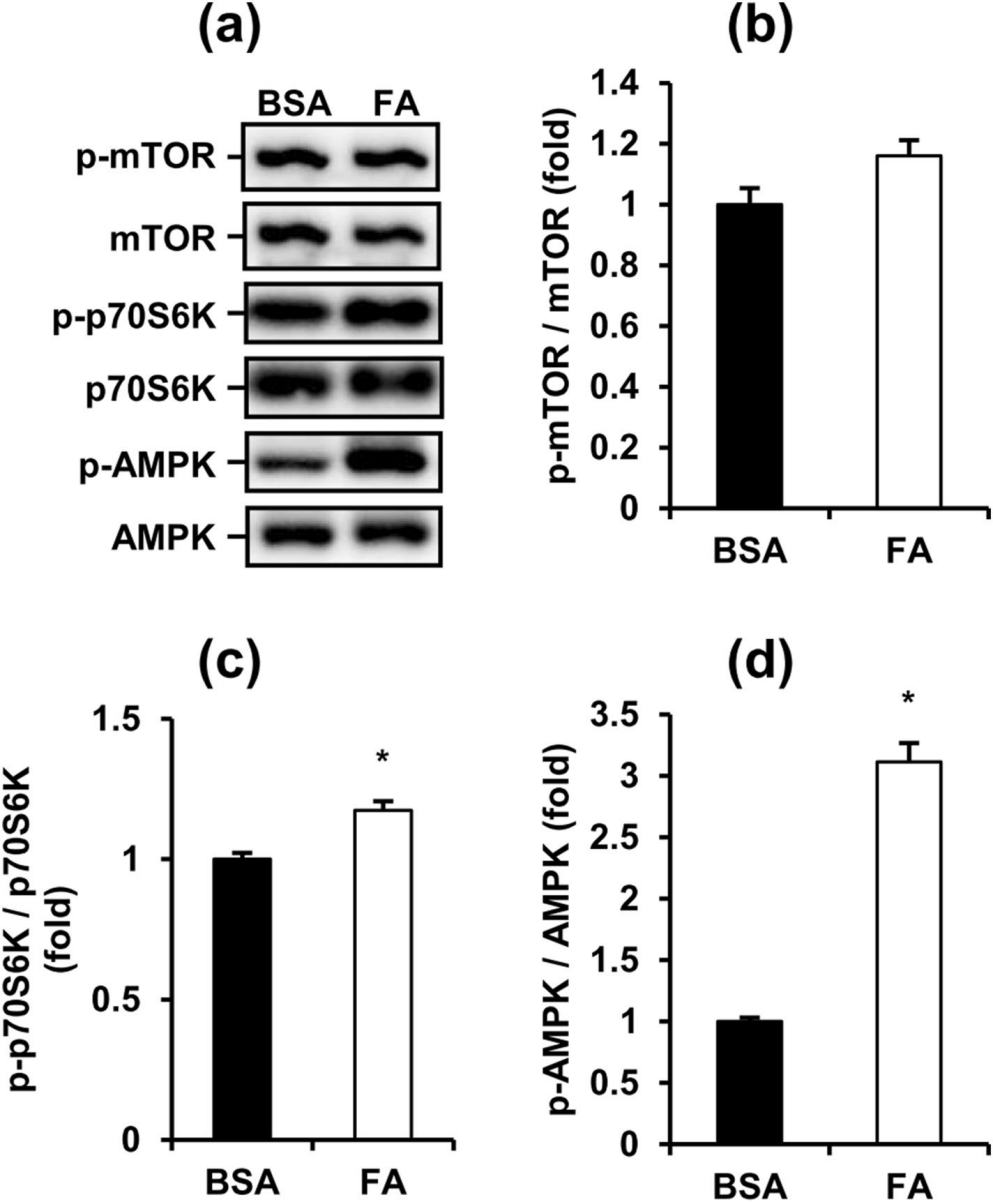
Fatty acids simultaneously activate the inhibition and induction signaling of early-stage autophagy in MA-10 cells. (**a**) Representative blots of mTOR and AMPK signaling in FA-induced MA-10 cells are shown. (**b**), (**c**), (**d**) Quantification of blots normalized by β-actin are shown as means ± SEM (n = 3). *P < 0.05.

To further investigate whether FAs also regulated the early-stage of autophagy, we next analyzed AMPK/mTOR/ULK1 signaling, which is the initiation indicator of autophagy in MA-10 cells. Compared with the BSA control group, the phosphorylation of mTOR (Ser2448) showed no significant difference but the phosphorylation of p70S6K (Thr389) showed a significant increase in FA-treated MA-10 cells (Fig. 4a, b, c). This pattern showed that mTOR (Ser2448) regulates inhibition of the ULK1 complex, which is essential to initiate autophagy in MA-10 cells activated under FA treatment. Additionally, the phosphorylation of AMPK (Thr172) also significantly increased after FA treatment for 48 h, indicating AMPK-regulated activation of the ULK1 complex in MA-10 cells was also induced (Fig. 4d). Collectively, these data demonstrated that FA simultaneously activates both the inhibition and induction signaling of early-stage autophagy in MA-10 cells.

### Rapamycin partially restores the FA-suppressed basal steroidogenesis of MA-10 cells

**Figure 5:**
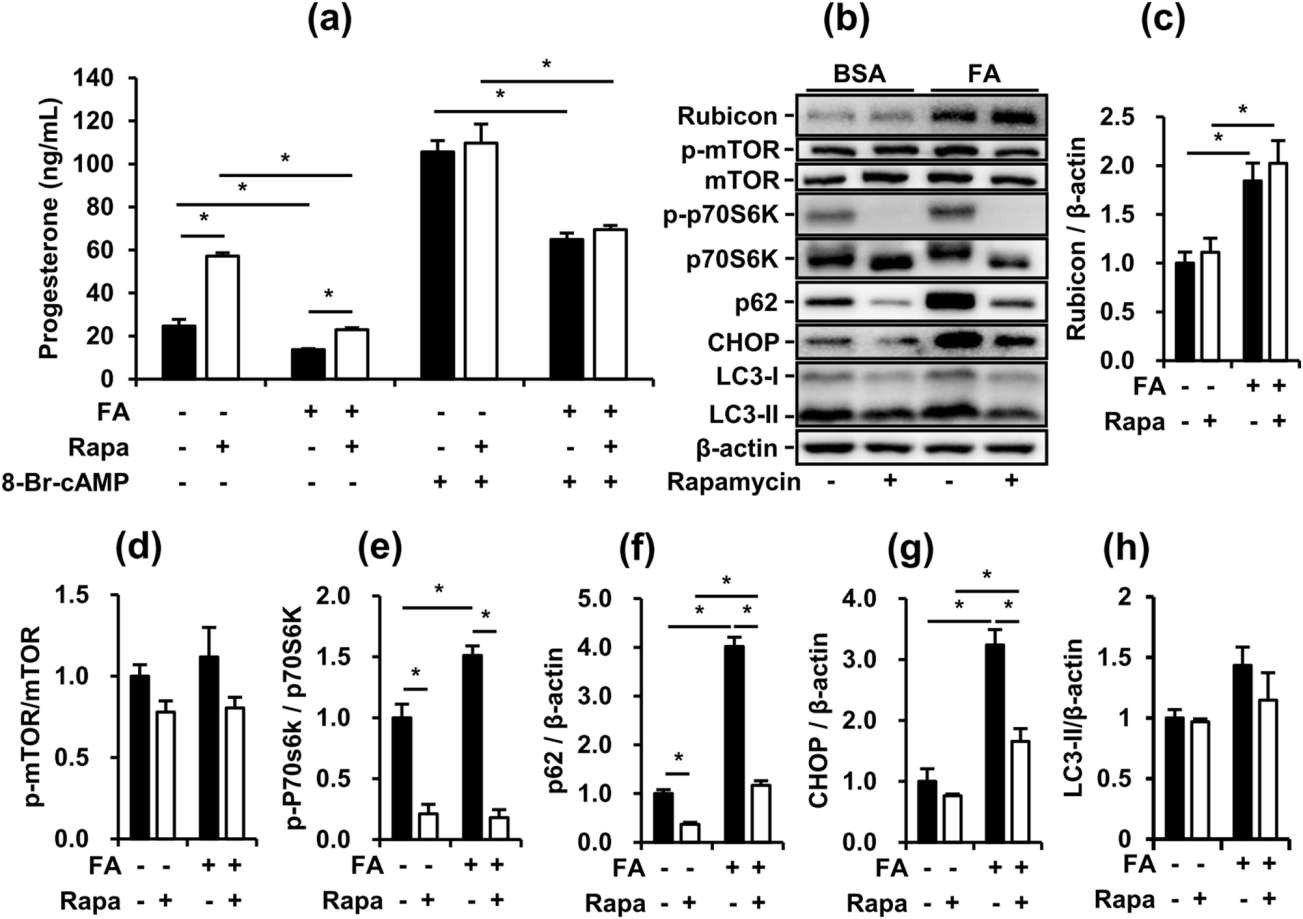
Rapamycin partially restores FA-suppressed basal steroidogenesis of MA-10 cells. After treatment with FA for 48 h and co-treatment with 10 nM rapamycin in the last 24 h, MA-10 cells were treated with or without 50 µM 8-Br-cAMP for 4 additional hours to induce steroidogenesis. (**a**) Progesterone levels in conditioned medium from the assigned groups of MA-10 cells. (**b**) Representative blots of autophagy and ER stress markers in cultured MA-10 cells are shown. (**c**), (**d**), (**e**), (**f**), (**g**), (**h**) Quantifications normalized by β-actin are shown as means ± SEM (n = 3). *P < 0.05.

To specifically clarify the effects of the induction of early-stage autophagy on FA-treated MA-10 cells, we applied rapamycin, a classic inhibitor of mTOR complex 1 (mTORC1), to potently activate the initiation of autophagy. Rapamycin increased P4 secretion from MA-10 cells in the basal state with and without FA. However, although rapamycin significantly activated early-stage autophagy, rapamycin had no more beneficial effects on P4 secretion from MA-10 cells under 8-Br-cAMP stimulation (Fig. 5a, b). To further understand how rapamycin affected the steroidogenesis of MA-10 cells, we analyzed the protein expression of mTOR signaling mediators, p62, LC3, Rubicon and CHOP in MA-10 cells under rapamycin treatment with or without FA (Fig. 5b). The phosphorylation of mTOR (Ser2448) showed no significant difference under rapamycin treatment, but the phosphorylation of p70S6K (Thr389) was significantly downregulated by rapamycin in MA-10 cells (Fig. 5d, e). Under rapamycin treatment, FA-upregulated p62 expression was partially decreased but the expression of LC3-II showed no difference in MA-10 cells (Fig. 5f, h). Rapamycin did not change FA-upregulated expression of Rubicon in MA-10 cells (Fig. 5c). Notably, although rapamycin suppressed the expression of p62 and CHOP upregulated by FA in MA-10 cells, FA-suppressed P4 secretion from MA-10 cells in the 8-Br-cAMP-stimulated state showed no improvement (Fig. 5a, f, g). These data indicated that rapamycin-activated autophagy restored FA-suppressed P4 secretion from MA-10 cells in the basal state but demonstrated no recovery effects on the maximum capacity of steroidogenesis under FA challenge. However, considering the diverse regulation of mTORC1 in cellular function, our data also suggest that rapamycin-stimulated signaling may share some of the pathways with 8-Br-cAMP-stimulated signaling in P4 secretion from MA-10 cells.

### Inhibition of autophagy suppresses both basal and stimulated steroidogenesis of MA-10 cells

**Figure 6:**
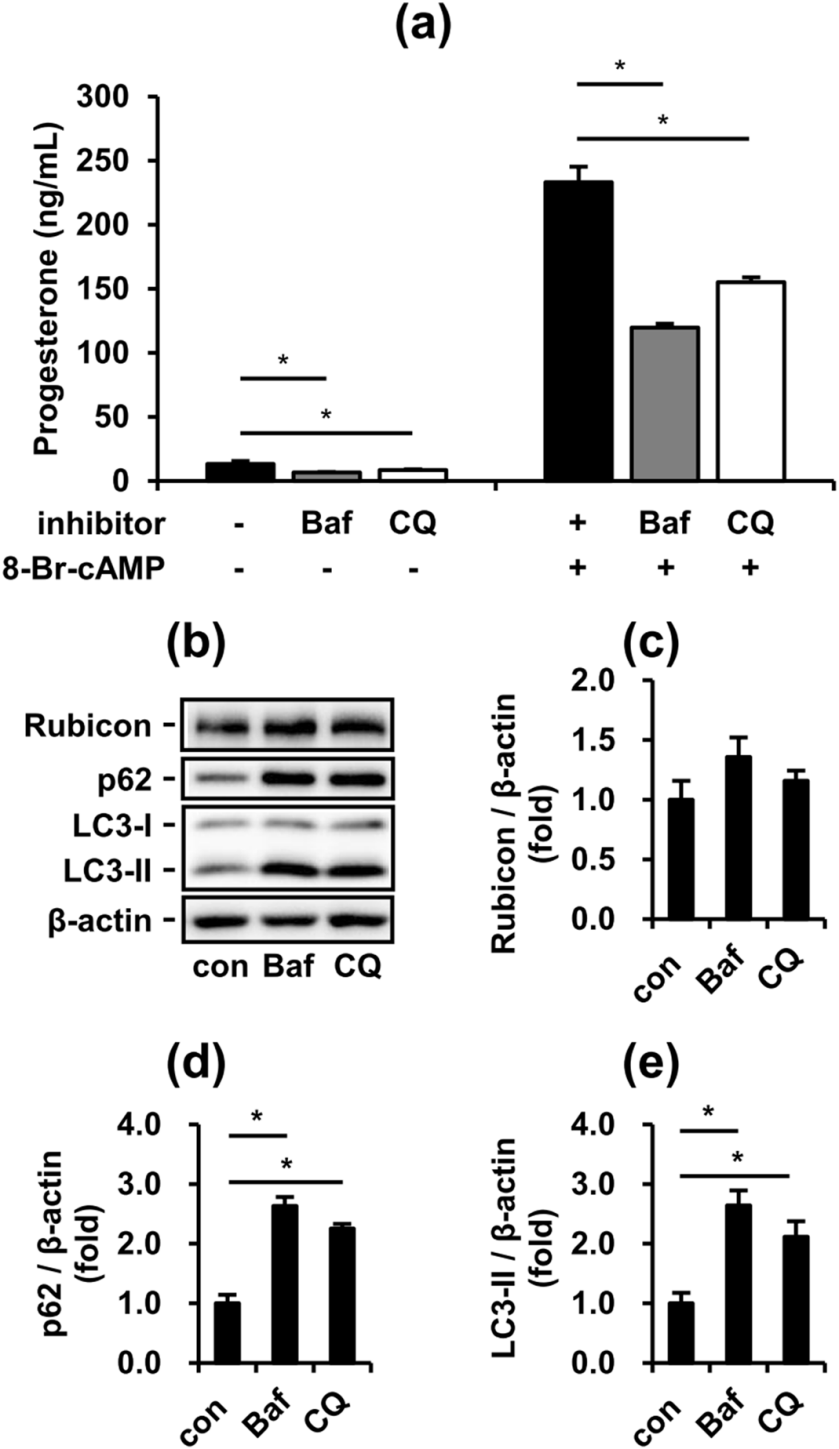
Inhibition of autophagy suppresses both basal and stimulated steroidogenesis of MA-10 cells. To further investigate the effects of inhibited autophagy in MA-10 cells, we applied two late-stage autophagy inhibitors, bafilomycin A1 (Baf) and chloroquine (CQ). After treatment with or without 50 nM Baf and 20 µM CQ for 24 h, the conditioned medium and cell lysates from cultured MA-10 cells were collected for further analysis. (**a**) Progesterone levels in condition medium from the assigned groups of MA-10 cells. (**b**) Representative blots of autophagy markers in cultured MA-10 cells. (**c**), (**d**), (**e**) Quantifications normalized by β-actin are shown as means ± SEM (n = 4). *P < 0.05.

After checking the effects of rapamycin on FA-suppressed steroidogenesis from MA-10 cells, we next checked whether blocking the late-stage of autophagy impacted P4 secretion from MA-10 cells. We applied two inhibitors of late-stage autophagy, bafilomycin A1 (Baf) and chloroquine (CQ), to MA-10 cells. Baf and CQ inhibit the maturation of autolysosome by interfering with the fusion between autophagosomes and lysosomes and acidification of autolysosomes. As expected, both Baf and CQ significantly accumulated p62 and LC3-II in MA-10 cells (Fig. 6b, d, e), representing common profiles of late-stage autophagy inhibition. Both Baf and CQ suppressed basal and 8-Br-cAMP-stimulated P4 secretion from MA-10 cells (Fig. 6a, b). Notably, both Baf and CQ exhibited no effect on the expression of the endogenous late-stage autophagy inhibitor Rubicon (Fig. 6b, c). Our data demonstrated that chemical inhibition of late-stage autophagy exerts similar inhibitory effects to FAs on P4 secretion from MA-10 cells.

### Rubicon plays an indispensable role in maintaining and regulating the steroidogenesis of MA-10 cells, independent of the inhibition of late-stage autophagy

**Figure 7:**
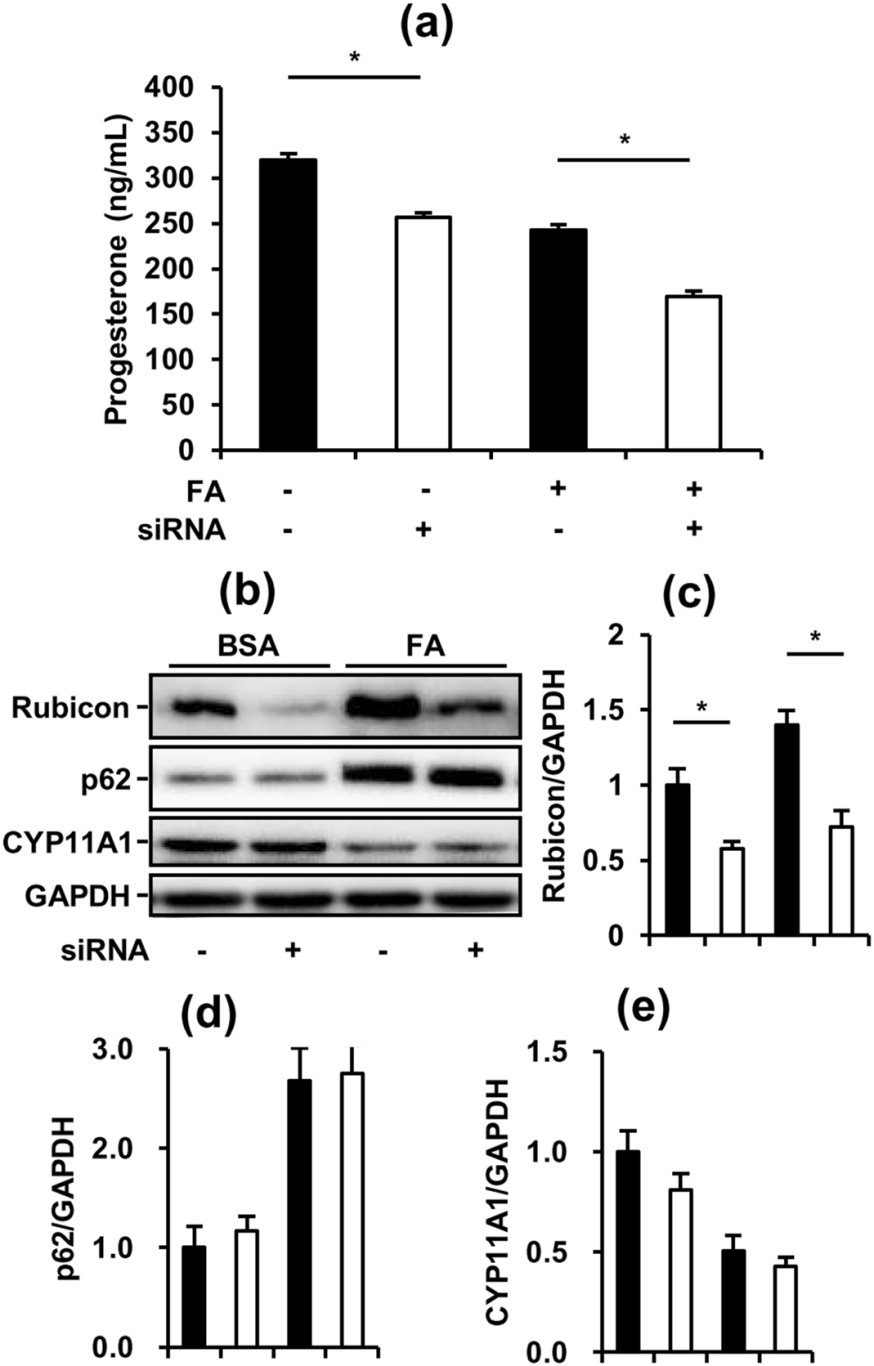
Rubicon plays an indispensable role in maintaining and regulating the steroidogenesis of MA-10 cells, independent of the inhibition of late-stage autophagy. To further check the effects of late-stage autophagy inhibition on FA-suppressed steroidogenesis, we applied siRNAs to knock down Rubicon. After transfection with or without 10 nM *Rubcn* siRNAs for 72 h and treatment with 1% BSA or 1200 µM fatty acids (0.6 mM PA mixed with 0.6 mM OA) in the last 48 h, the conditioned medium and cell lysates from cultured MA-10 cells were collected for further analysis. (**a**) Progesterone levels in condition medium from the assigned groups of MA-10 cells. (**b**) Representative blots of autophagy and steroidogenesis markers in cultured MA-10 cells. (**c**), (**d**), (**e**) Quantifications normalized by GAPDH are shown as the means ± SEM (n = 3). *P < 0.05.

To further understand whether the upregulation of Rubicon by FAs was the major factor of suppressed P4 secretion from MA-10 cells, we transfected siRNAs targeting *Rubcn* to downregulate Rubicon expression. Unexpectedly, P4 secretion from MA-10 cells was significantly decreased by Rubicon knockdown not only in the basal state but also in the 8-Br-cAMP-stimulated state (Fig. 7a). To validate the knockdown efficiency, we also measured the expression of Rubicon and p62. In both states, Rubicon was significantly knocked down but p62 was not changed by Rubicon siRNAs in MA-10 cells. Furthermore, we found that CYP11A1 expression showed no significant difference after Rubicon knockdown (Fig. 7b, c, d, e). Collectively, our data indicated that both basal and 8-Br-cAMP-stimulated P4 secretion from MA-10 cells may be mostly dependent on CYP11A1 expression. Additionally, Rubicon could play unknown physiological roles in the steroidogenesis of MA-10 cells independent of late-stage blockers of autophagy.

## Discussion

Although many factors are involved, male obesity has recently been considered a prominent indicator of infertility, and obese men have lower circulating testosterone than men with an optimal BMI^27,28^. Obese patients have elevated levels of FFAs in their blood causing toxicity to different cell types^29–31^. As reported by Meikle et al., a higher FFA concentration suppressed luteinizing hormone (LH)-stimulated testosterone production from isolated mouse Leydig cells^5^. Additionally, many studies have shown that the excess cellular lipid content disrupts autophagy, leading to dysfunction in many cell types^25,26^. Thus, we hypothesized that FA might impair steroid production by dysregulating autophagy in Leydig cells. This study characterized FA impairment of steroidogenesis in Leydig cells and clarified the role of autophagy in this FA-induced model.

To study the molecular mechanisms in FA-affected Leydig cells more deeply, we employed the mouse Leydig cell line MA-10 for further experiments. To mimic the plasma FFA profile in obese mice, MA-10 cells were treated with an FA mixture containing equal concentrations of oleic acid (OA) and palmitic acid (PA) at different dosages for 48 h. Notably, because of the lack of the enzyme CYP17A1, which converts progesterone to androstenedione, MA-10 cells lack the normal capability to produce testosterone. Therefore, we measured P4 production as an indicator for the early-stage steroidogenesis of Leydig cells in this study. Additionally, to test the capacity of steroidogenesis, MA-10 cells were treated with or without 50 µM 8-Br-cAMP for 4 h. To mimic the plasma FFAs of mice during obesity, a 0.8- to 1.2-mM FA mixture containing the same amount of OA and PA was added to the culture medium. The FA concentration and composition were similar to the plasma profile of obese mice^32^. In our MA-10 model, the 0.8-mM FA mixture significantly induced lipid accumulation but had no effects on progesterone (P4) production in both the basal and 8-Br-cAMP-stimulated states (Fig. 1). Additionally, the 1.2 mM FA mixture further induced lipid accumulation and significantly inhibited P4 production in both states simultaneously. Next, we applied the 1.2-mM FA mixture and defined the groups treated with and without 8-Br-cAMP as the basal and stimulated states, respectively, in subsequent experiments (Fig. 1b). Besides, we tried to apply the long-term exposure of FAs to primary mouse Leydig cells. Consistent to the findings from MA-10 model, the progesterone and testosterone levels significantly decreased with increasing FA concentration in both the basal and 8-Br-cAMP-stimulated states (supplementary figure S7a). However, the GAPDH expression were significantly downregulated by FA mixtures in both basal and 8-Br-cAMP-stimulated states, indicating long-term exposure of FAs induced strong apoptosis of primary Leydig cell even under rational concentration during obesity (supplementary figure S7b). Considering our results from MA-10 model, these data suggest that the progesterone and testosterone decrease caused by high concentration of FAs may be due to the decrease of precursors including P4. However, it is difficult to exclude that the testosterone decrease of primary Leydig cells is due to strong apoptosis. Therefore, we did not use primary mouse Leydig cells for subsequent analysis.

The steroidogenic process depends on a series of enzymatic steps; thus, we first examined the protein expression of steroidogenic enzymes in FA-treated MA-10 cells. Our data showed that the long-term exposure of FAs significantly changed steroidogenic signaling mediators, including p-CREB, cleaved StAR, and CYP11A1 (Fig. 2). Although previous studies have shown that CREB phosphorylation is highly correlated with the recruitment of CREB binding protein to the StAR promoter^33^, in our MA-10 model, FA significantly decreased CREB phosphorylation but increased level of cleaved StAR. Additionally, FA downregulated the protein expression of CYP11A1 in MA-10 cells. These findings first revealed the possible mechanisms of steroidogenesis under long-term exposure to FAs and their involvement in impaired steroidogenesis of Leydig cells. To determine the major impairment, we tried to restore the FA-suppressed steroidogenesis by applying 22R-OHC and P5 to MA-10 cells in both the basal and stimulated states. However, 22R-OHC could not recover FA-suppressed steroidogenesis in both states (Fig. 2e). 22R-OHC is a membrane-permeable cholesterol that directly enters the inner mitochondrial membrane without the help of StAR. These results illustrated that MA-10 cells had sufficient substrates for CYP11A1 enzyme under FA exposure, a finding that was not consistent with a previous study^5^. This study demonstrated the short-term effects of FAs (3 h) on testosterone secretion from primary Leydig cells. We consider the previous model using primary Leydig cell only mimic the transient increase of blood lipids after a meal but not long-term exposure of FFA during obesity. This difference may be due to the different FA exposure times, differences between MA-10 and primary Leydig cells and differences in steroid production indicators. Strikingly, in the MA-10 model, pregnenolone (P5) mitigated FA-suppressed steroidogenesis in the basal state and totally recovered FA-suppressed steroidogenesis in the 8-Br-cAMP-stimulated states (Fig. 2e). P5 is the metabolic product of CYP11A1 and a substrate for 3β-HSD. Collectively, our data indicated that the major impairment in FA-suppressed steroidogenesis of MA-10 cell was deficient CYP11A1 activity.

To determine whether autophagy imbalance was involved in FA-mediated steroidogenesis, we analyzed autophagy and ER stress markers in FA-treated MA-10 cells. FA upregulated Rubicon, p62 and CHOP but had no effects on the expression of Beclin 1 and LC3-II in MA-10 cells (Fig. 3). These data indicated that long-term FA exposure not only promoted the blockade of late-stage autophagy but also accumulated improperly folded proteins in the ER. To further elucidate the autophagic state in FA-treated MA-10 cells, we analyzed early-stage markers of autophagy. FA simultaneously increased phosphorylation of p70S6K and AMPK (Fig. 4). Phosphorylation of p70S6K (Thr389) is a downstream indicator of mTOR-activated inhibition of autophagy initiation. Conversely, phosphorylation of AMPK (Thr172) is an activating indicator of autophagy initiation. Interestingly, our data showed that long-term FA exposure simultaneously inhibited and activated the initiation of autophagy, representing a very special pattern compared with published data^22,34–36^. In other cell types, mTOR-inhibited and AMPK-activated initiation of autophagy usually show opposite trends under the same stimulation. Considering the higher fold of AMPK (Thr172) phosphorylation than p70S6K (Thr389) phosphorylation and p62 upregulation, we suggest that FA had a net increase of early-stage autophagy in MA-10 cells.

Although our data showed that FA inhibited both early-stage and late-stage autophagy and activated early-stage autophagy, the major regulator of steroidogenesis during FA challenge was difficult to clarify. To exclude the roles of early-stage and late-stage autophagy in FA-suppressed steroidogenesis, we treated the MA-10 model with rapamycin to induce early-stage autophagy. Rapamycin is a specific inhibitor of mTORC1, which is a natural blocker of early-stage autophagy. Our data demonstrated that rapamycin strongly activated early-stage autophagy, consistent with previous study findings (Fig. 5b). Additionally, we found that rapamycin significantly increased the steroidogenesis of MA-10 in the basal state but not in the 8-Br-cAMP-stimulated state (Fig. 5a). These data suggest that rapamycin-activated initiation of autophagy may be involved in the pathway of 8-Br-cAMP-stimulated steroidogenesis independent of FA suppression. The involvement of rapamycin in 8-Br-cAMP-stimulated steroidogenesis may be derived from the mTOR-regulated phosphorylation of PKA^37^. Additionally, cleaved StAR induced by 8-Br-cAMP showed positive correlation with P4 secretion in the basal state (Fig. 2a, c). However, more data are required to elucidate the involvement of rapamycin in 8-Br-cAMP-stimulated steroidogenesis. Furthermore, to mimic the inhibition of late-stage autophagy under FA challenge, we applied two inhibitors of late-stage autophagy to MA-10 cells. Both Baf and CQ inhibited late-stage autophagy in MA-10 cells and suppressed steroidogenesis in both the basal and 8-Br-cAMP-stimulated states. Consistent with the FA-inducing model, these data indicated that the inhibition of late-stage autophagy significantly suppressed the steroidogenesis of MA-10 cells. Thus, we further attempted to restore FA-suppressed steroidogenesis by transfecting siRNA targeting the *Rubcn* gene. However, unexpectedly, when Rubicon was significantly silenced, the steroidogenesis of MA-10 cells was further suppressed in addition to FA-suppressed steroidogenesis (Fig. 7). These data indicated that Rubicon not only inhibited late-stage autophagy in MA-10 cells but also had indispensable functions for maintaining the integrity of steroidogenesis. Previous studies have shown that Rubicon interacts with other proteins via multiple functional domains to mediate physiological functions^38,39^. Under different stimuli, Rubicon is recruited by different complexes, such as the class III PI3K complex and NOX2 complex, to participate in canonical and noncanonical autophagy and the inflammatory response^40^. Here, our study exhibited a novel potential of Rubicon for maintaining the steroidogenesis of Leydig cells.

In summary, our study demonstrated that the FA-suppressed steroidogenesis of MA-10 cells could be almost completely recovered by P5 treatment, indicating that CYP11A1 deficiency was the primary defect in this model. Based on the rapamycin experiments, we suggest that FA-suppressed steroidogenesis is independent of early-stage autophagy. Our data also highlight that inhibition of late-stage autophagy including FA-upregulated Rubicon leads to impaired steroidogenesis in MA-10 cells. Additionally, our results show that Rubicon has regulatory functions in steroidogenesis independent of blockers of late-stage autophagy. In conclusion, this study provides new insights into obesity-related impaired steroidogenesis and novel targets with research potential in steroidogenic cells.

## Methods

### Cell culture

The MA-10 mouse Leydig cells were purchased from American Type Culture Collection (ATCC) and cultured in DMEM/F12 medium (Sigma-Aldrich; D8900) supplemented with 15% horse serum (Gibco), 1% penicillin–streptomycin (Gibco), 20 mM HEPES and 1.2 g/L of sodium bicarbonate. After pre-coating 0.1% gelatin on the dishes and plates for 4 h, the cells were maintained at 37℃ with 5% CO_2_ in a humidified incubator. Palmitic acid (PA) and oleic acid (OA) were separately dissolved in pure ethanol (100 mM) and diluted to a working concentration with the culture medium containing 1% BSA to conjugate fatty acids. To stimulate steroidogenesis, the cells were treated with 50 μM 8-Br-cAMP (Tocris) for 4 h, and then the conditioned medium was collected for further measurements. We treated the cells with 10 µg/mL of 22(R)-hydroxycholesterol (22R-OHC; Sigma) to assay CYP11A1 activity and 1 µg/mL of pregnenolone (Sigma) to assay 3β-HSD activity for 4 h and measured the progesterone production. To modulate autophagic activities, the cells were treated with 50 nM bafilomycin (Tocris), 20 μM chloroquine (Sigma-Aldrich), and different concentrations of rapamycin (Tocris) for 24 h before analysis. For the knockdown experiments, SMARTpool specific siRNAs (Dharmacon) were reversely transfected using Lipofectamine RNAiMAX Transfection Reagent (Thermo).

### Animals

Male ICR mice obtained from the Laboratory Animal Center of the College of Medicine at National Taiwan University were maintained under a 12-h light and 12-h darkness cycle, 40–60% relative humidity and ad libitum water and feed. Primary Leydig cells were isolated from 12- to 16-week-old mice followed as description in supplementary methods. All animal experiments were approved by Institutional Animal Care and Use Committee of National Taiwan University and performed according to the Guide for the Care and Use of Laboratory Animals and ARRIVE guidelines.

### Enzyme-linked immunosorbent assay (ELISA)

The concentrations of progesterone in the culture medium were determined using a competitive ELISA, as previously described^41^. Briefly, 96-well plates were coated with mouse anti-progesterone antibodies. The standard solutions and diluted medium (50 μl) were mixed with the progesterone-11-HS-HRP solution (150 μl) in the plates. All the standards and samples were assayed in duplicate. After 25 min of incubation, followed by rinsing, color development was performed in 0.1 M phosphate buffer (pH 6.0) containing 3.7 mM o-phenylenediamine (200 μl) for 20 min. The reactions were stopped by adding 50 μl of 8 N sulfuric acid. The optical densities were determined by the absorbance at a wavelength of 490 nm. The background signals were detected at a wavelength of 630 nm. The progesterone concentration in the medium was calculated based on the standard curve. In the progesterone assay, the intra- and inter-assay coefficients of variation are 4.6% and 6.9%.

### Lipid accumulation assay

To quantify intracellular lipids, Nile Red fluorescent staining was performed on the MA-10 cells. After 4% paraformaldehyde fixation for 15 min, cultured MA-10 cells in black 96-well plates were stained using 10 μg/ml of Nile Red (30 min; excitation: 485 nm; emission: 580 nm) and 5 μg/ml of Hoechst 33,342 (2 min; excitation: 355 nm; emission: 465 nm) in PBS. The fluorescent signals from MA-10 cells were directly read in the plate using a Synergy H1 hybrid multi-mode reader (BioTek Instruments Inc.). The intracellular lipids (Nile Red signal) normalized by the cell number (Hoechst 33,342 signal) from each sample was finally identified as lipid accumulation for further statistical analysis.

### Western blot analysis

To harvest the total protein in MA-10 cells, the cultured cells were rinsed once with PBS and then lysed with 1 × Laemmli buffer (2% sodium dodecyl sulfate [SDS], 5 mM DTT, 10% glycerol, 0.002% bromophenol blue, and 63 mM Tris HCl at pH = 6.8). The collected lysates were heated at 98℃ for 7 min and separated by 6–13% sodium dodecyl sulfate–polyacrylamide gel electrophoresis (SDS-PAGE). The separated proteins were then transferred from the gel onto polyvinylidene difluoride membranes (1,620,177; Bio-Rad). After protein transfer, the PVDF membranes were blocked with 5% non-fat milk in TBS-T (20 mM Tris, 150 mM NaCl, 0.1% Tween-20) for 1 h at room temperature, followed by hybridization with primary antibodies at 4℃ overnight. Next, after washing with TBS-T three times for 10 min, the membranes were incubated with species-specific secondary antibodies (in 5% non-fat milk in TBS-T) for 1 h. Finally, the membranes were washed with TBS-T three times for 10 min and then incubated with ECL substrates (RPN2235; GE Healthcare) using the Bio-Rad ChemiDoc Imaging System. Bio-Rad Image Lab software was used for blot quantification. The normalization of target protein was conducted relative to internal controls. The antibodies used in this study included anti-p-AMPKα (1000×; #2535; Cell Signaling Technology), anti-AMPKα (1000×; #2532; Cell Signaling Technology), anti-Beclin-1 (1000×; #3495; Cell Signaling Technology), anti-β-actin (2000×; sc-47778; Santa Cruz Biotechnology), anti-CHOP (1000×; #5554; Cell Signaling Technology), anti-CYP11A1 (1000×; #14217; Cell Signaling Technology), anti-p-CREB (1000×; #9198; Cell Signaling Technology), anti-CREB (1000×; #9197; Cell Signaling Technology), anti-GAPDH (2000×; #2118; Cell Signaling Technology), anti-LC3 (1000×; #2775; Cell Signaling Technology), anti-p-mTOR (1000×; #2983; Cell Signaling Technology), anti-mTOR (1000×; #2983; Cell Signaling Technology), anti-SQSTM1/p62 (5000×; ab109012; Abcam), anti-p-p70S6K (1000×; #9234; Cell Signaling Technology), anti-p70S6K (1000×; #2708; Cell Signaling Technology), anti-Rubicon (1000×; #8465; Cell Signaling Technology), goat anti-rabbit IgG-HRP (5000×, sc-2004, Santa Cruz Biotechnology; 2500×, #31460, Thermo Fisher Scientific) and goat anti-mouse IgG-HRP (5000×, sc-2005, Santa Cruz Biotechnology; 2500×, #62–6520, Thermo Fisher Scientific).

### Statistical analysis

All the cell experiments were replicated at least for 3 times. The quantitative data were represented as mean ± standard error of the mean (SEM). Statistically significant differences (*P* < 0.05) were determined by Student’s t-test or one-way ANOVA followed with Duncan’s multiple range test which were performed by SigmaPlot software (version 12.0, Systat Software).

## Supplementary Information

Supplementary Information.

## Data Availability

All the data generated or analyzed during this study are included in this published article (and its Supplementary Information files).
