# Supplementary material for: Fatty acids suppress the steroidogenesis of the MA-10 mouse Leydig cell line by downregulating CYP11A1 and inhibiting late-stage autophagy
Source: Sci Rep. 2021 Jun 15;11:12561. doi: 10.1038/s41598-021-92008-2 (PMC8206377; doi:10.1038/s41598-021-92008-2)
Supplement: Supplementary file 1 — Supplementary Information. [file 41598_2021_92008_MOESM1_ESM.pdf]

Fatty acids suppress the steroidogenesis of the MA-10 mouse Leydig cell line by downregulating CYP11A1 and inhibiting late-stage autophagy

Chien Huang[1#], Hsiu-Ju Hsu[1#], Mu-En Wang[2], Meng-Chieh Hsu[3], Leang-Shin Wu[1], De-Shien Jong[1], Yi-Fan Jiang[4*], and Chih-Hsien Chiu[1*]

[1]Laboratory of Animal Physiology, Department of Animal Science and Technology, National Taiwan University, Taipei 10617, Taiwan
[2]Department of Pathology, Duke University School of Medicine, Durham, NC, USA
[3]Biochemistry Section, Surgical Neurology Branch, National Institute of Neurological Disorders and Stroke, National Institutes of Health, Bethesda, MD, USA
[4]Graduate Institute of Molecular and Comparative Pathobiology, School of Veterinary Medicine, National Taiwan University, Taipei 10617, Taiwan

[#]These authors contributed equally to this work.

***Corresponding Author: Chih-Hsien Chiu**
Laboratory of Animal Physiology, Department of Animal Science and Technology
National Taiwan University
No. 50, Lane 155, Section 3, Keelung Road, Taipei City 106, Taiwan
Tel.: +886-2-3366-4171; Fax: +886-2-3366-4070; Email: chiuchihhsien@ntu.edu.tw
***Co-corresponding Author: Yi-Fang Jiang**
[2]Graduate Institute of Molecular and Comparative Pathobiology, School of Veterinary Medicine
National Taiwan University
Rm. 104-1, No.1, Sec. 4, Roosevelt Road, Taipei City 106, Taiwan
Tel.: +886-2-3366-3765; Email: yfjiang@ntu.edu.tw

Supplementary Methods

Isolated mouse Leydig cell culture

The tunica albuginea of testis from 12- to 16-week-old mice was removed and the testicular contents were rinsed by Hank's balanced salt solution (HBSS). Then, washed contents were changed into centrifuge tube with additional 10 mL HBSS. The contents were dissociated by slowly shaking for 5 min. The dissociated solution was filtered by a strainer and the filtered solution were centrifuged at 300 g for 5 min. The Leydig cell were then resuspended in Medium 199 (Sigma-Aldrich) with 15 % horse serum. The crude Leydig cells were seeded in 10^5 cells/well on 24-well plate. Different dosages of OA and PA mixture were treated and 50 μM 8-Br-cAMP was co-treated to induce steroidogenesis. Conditioned medium and cell lysates were collected for further analysis.

Testosterone measurement

The ELISA procedure was described in main text. For testosterone analysis, testosterone antibody and HRP-conjugated testosterone (Cosmo Bio Co) were used in the assay. In the testosterone assay, the intra- and inter-assay coefficients of variation are 3.5 % and 6.7 %.

Supplementary Figure Legends

Supplementary Figure S1 The full-length blot of p-CREB, CREB, cleaved StAR, β-actin, CYP11A1, GAPDH presented in Figure 2 of the main text.

Supplementary Figure S2 The full-length blot of Rubicon, p62, Beclin-1, CHOP, LC3, β-actin presented in Figure 3 of the main text.

Supplementary Figure S3 The full-length blot of p-mTOR, mTOR, p-p70S6K, p70S6K, p-AMPK, AMPK presented in Figure 4 of the main text.

Supplementary Figure S4 The full-length blot of Rubicon, p-mTOR, mTOR, p-p70S6K, p70S6K, p62, CHOP, LC3, β-actin presented in Figure 5 of the main text.

Supplementary Figure S5 The full-length blot of Rubicon, p62, LC3, β-actin presented in Figure 6 of the main text.

Supplementary Figure S6 The full-length blot of Rubicon, p62, CYP11A1, GAPDH presented in Figure 7 of the main text.

Supplementary Figure S7 Fatty acids inhibit progesterone and testosterone synthesis of primary mouse Leydig cells. Primary mouse Leydig cells were treated with 1 % BSA, 0.8 mM to 1.2 mM fatty acid mixture for 48 hours and co-treated with or without 50 μM 8-Br-cAMP for 4 additional hours to induced steroidogenesis. Conditioned medium was collected to measure steroidogenesis. (a) The progesterone and testosterone levels in the conditioned medium. (b) Representative blots of GAPDH from cell lysates was shown and the quantifications of blots are shown as mean ± SEM (n=3). Different letters represent a significant difference between groups analyzed by one-way ANOVA followed with Duncan’s multiple comparisons ($P<0.05$).

Supplementary Figure S8 The full-length blot of GAPDH presented in Supplementary Figure S7.

Supplementary Figure S9 The effects of fatty acids on autophagy markers of primary mouse Leydig cells. Primary mouse Leydig cells were treated with 1 % BSA, 0.8 mM to 1.2 mM fatty acid mixture for 48 hours and co-treated with or without 50 μM 8-Br-cAMP for 4 additional hours to induce steroidogenesis. Representative blots of p62, LC3, ATG5 from cell lysates was shown (n=3).

Supplementary Figure S10 Fatty acids inhibit the steroidogenesis of MA-10 cells in response to different dose of hCG.
MA-10 cells were treated with 1 % BSA and 1.2 mM fatty acid mixture for 48 hours and co-treated with different dose of hCG for 4 additional hours to induce steroidogenesis. The progesterone levels in the conditioned medium are represented as means ± SEM (n = 3). *$P<0.05$

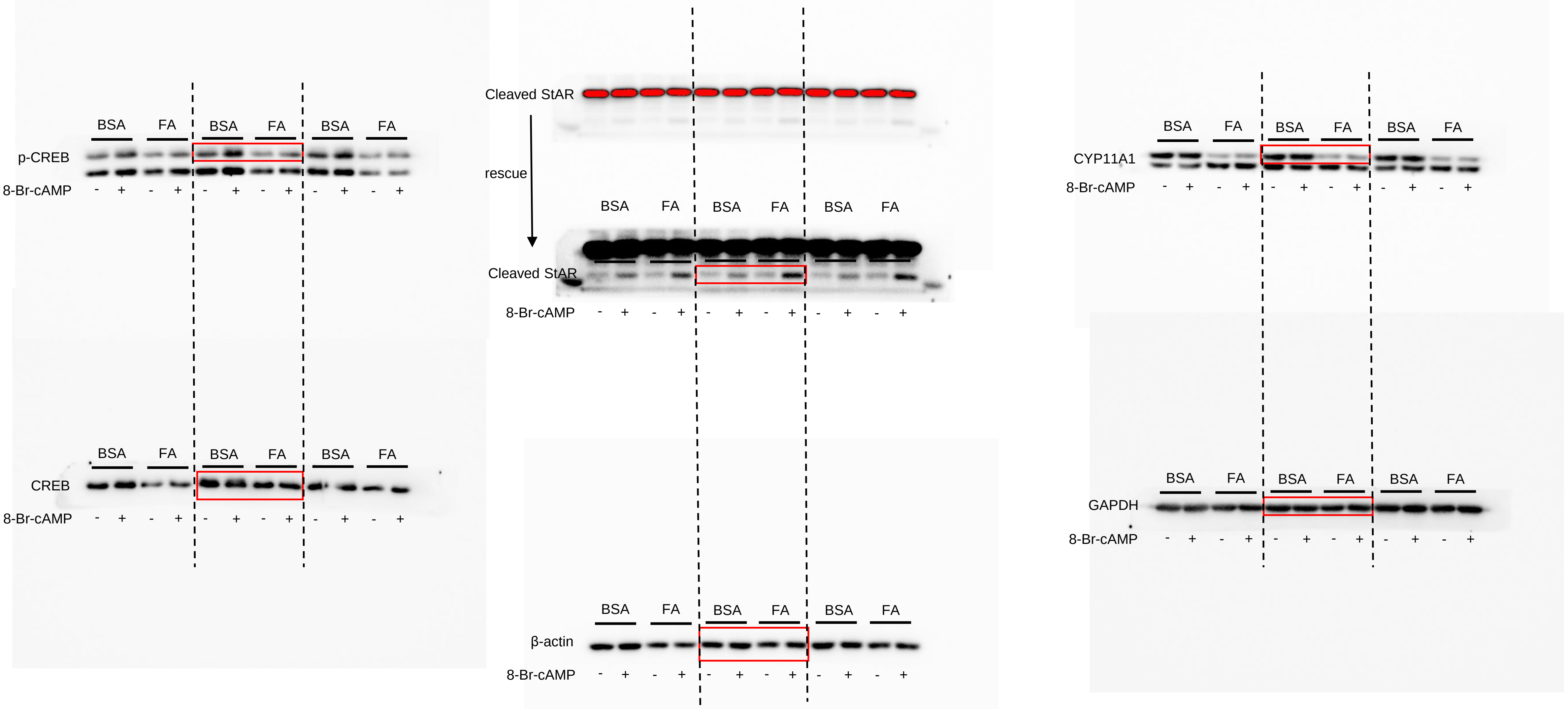

Supplementary Figure S1

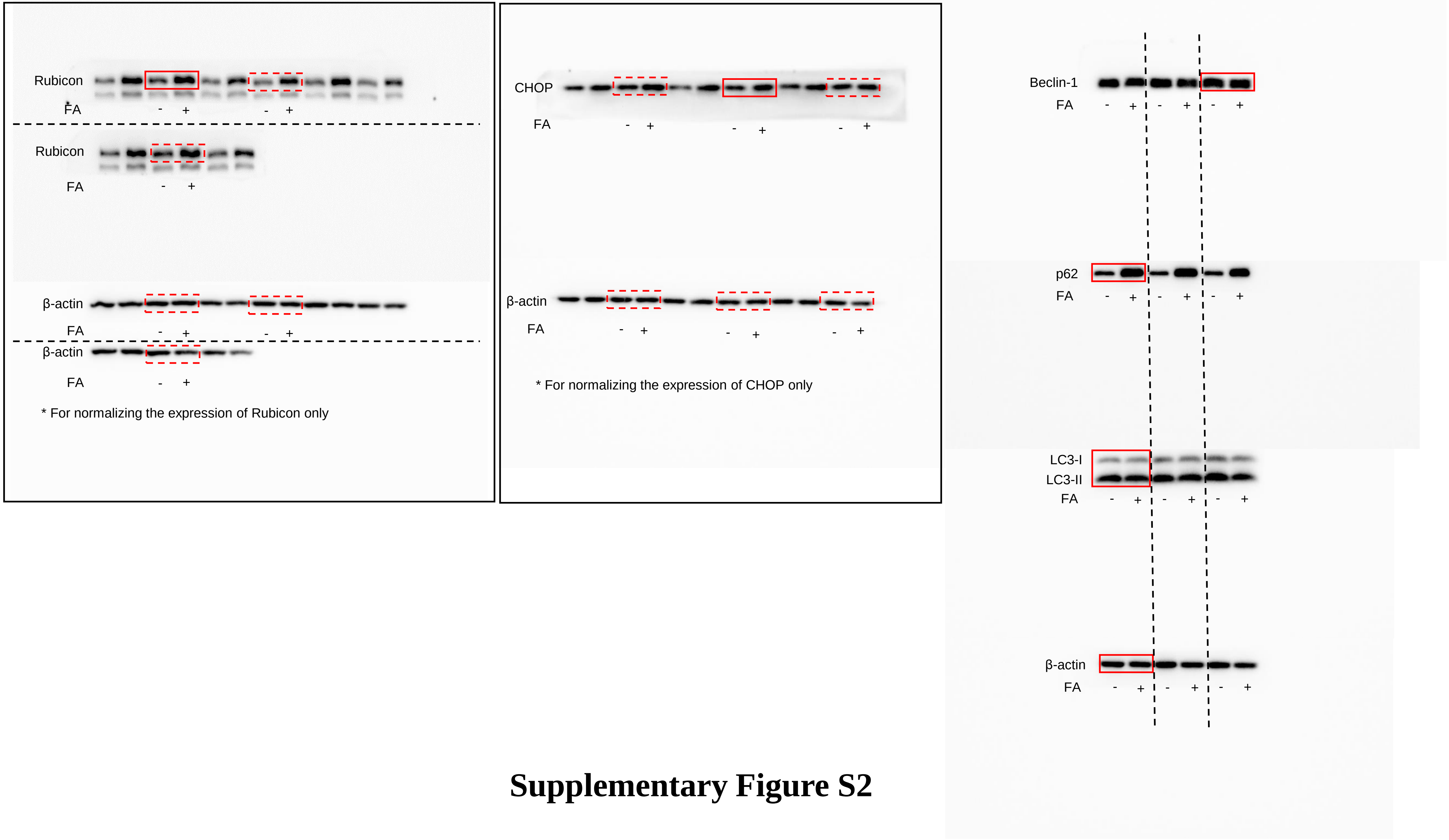

Supplementary Figure S2

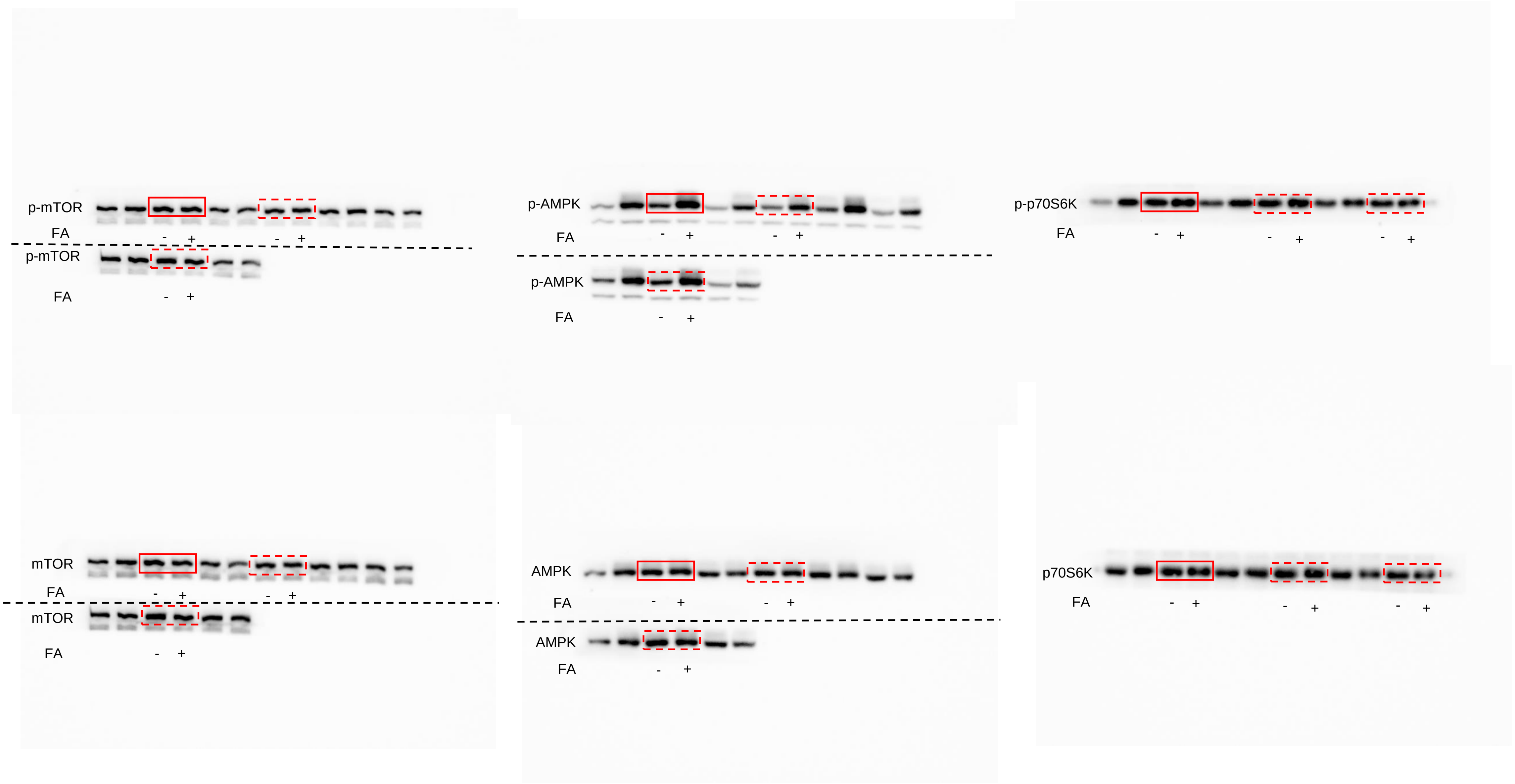

Supplementary Figure S3

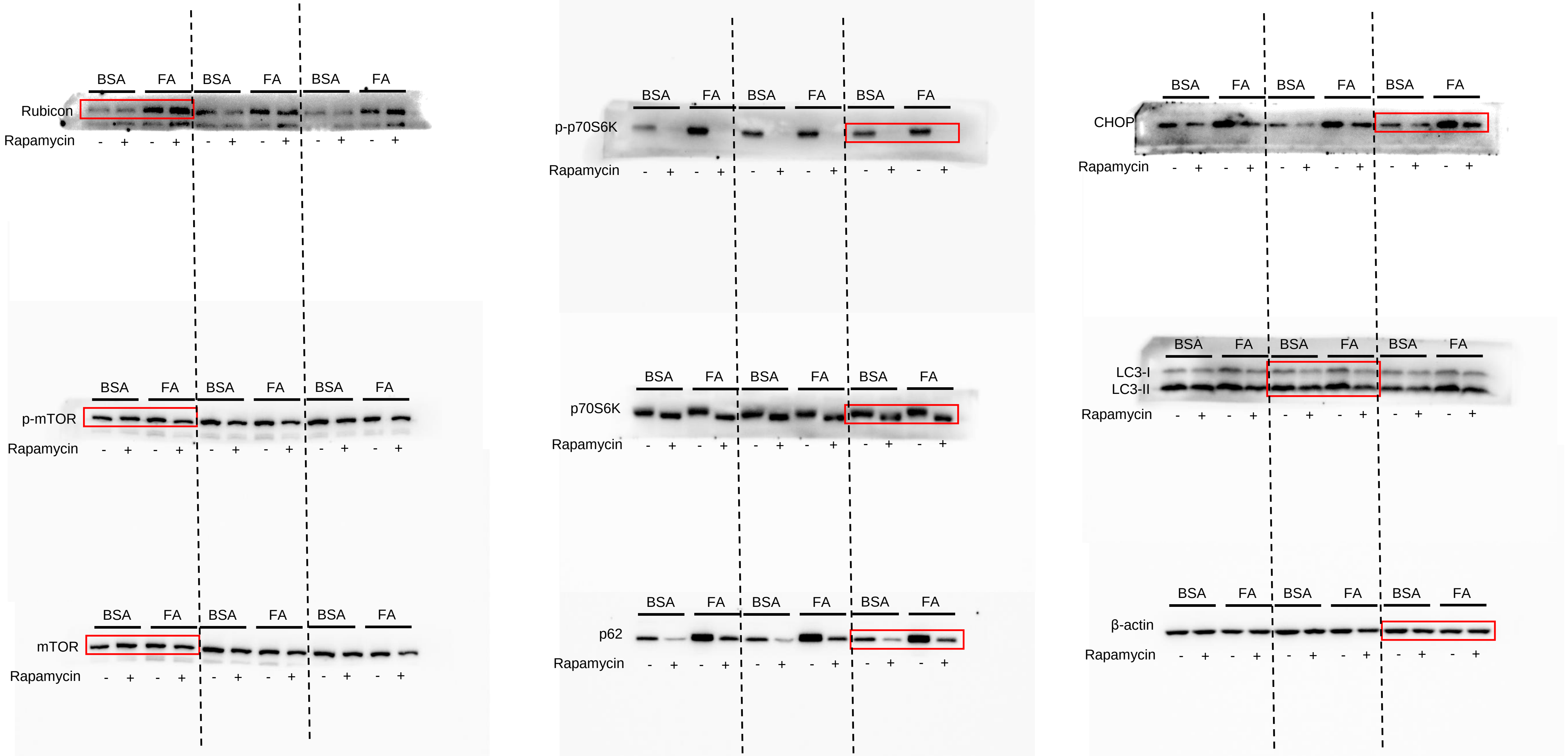

Supplementary Figure S4

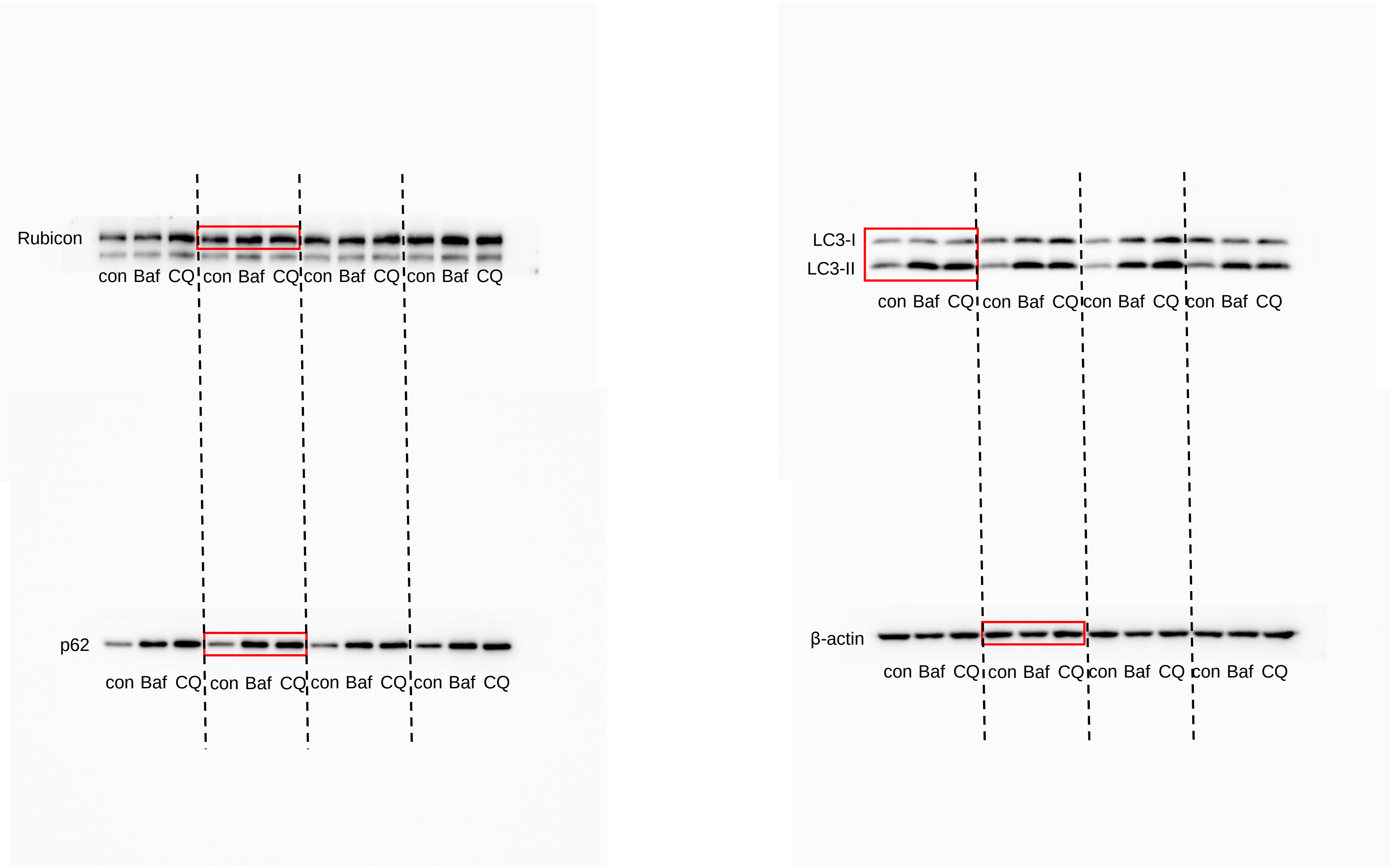

Supplementary Figure S5

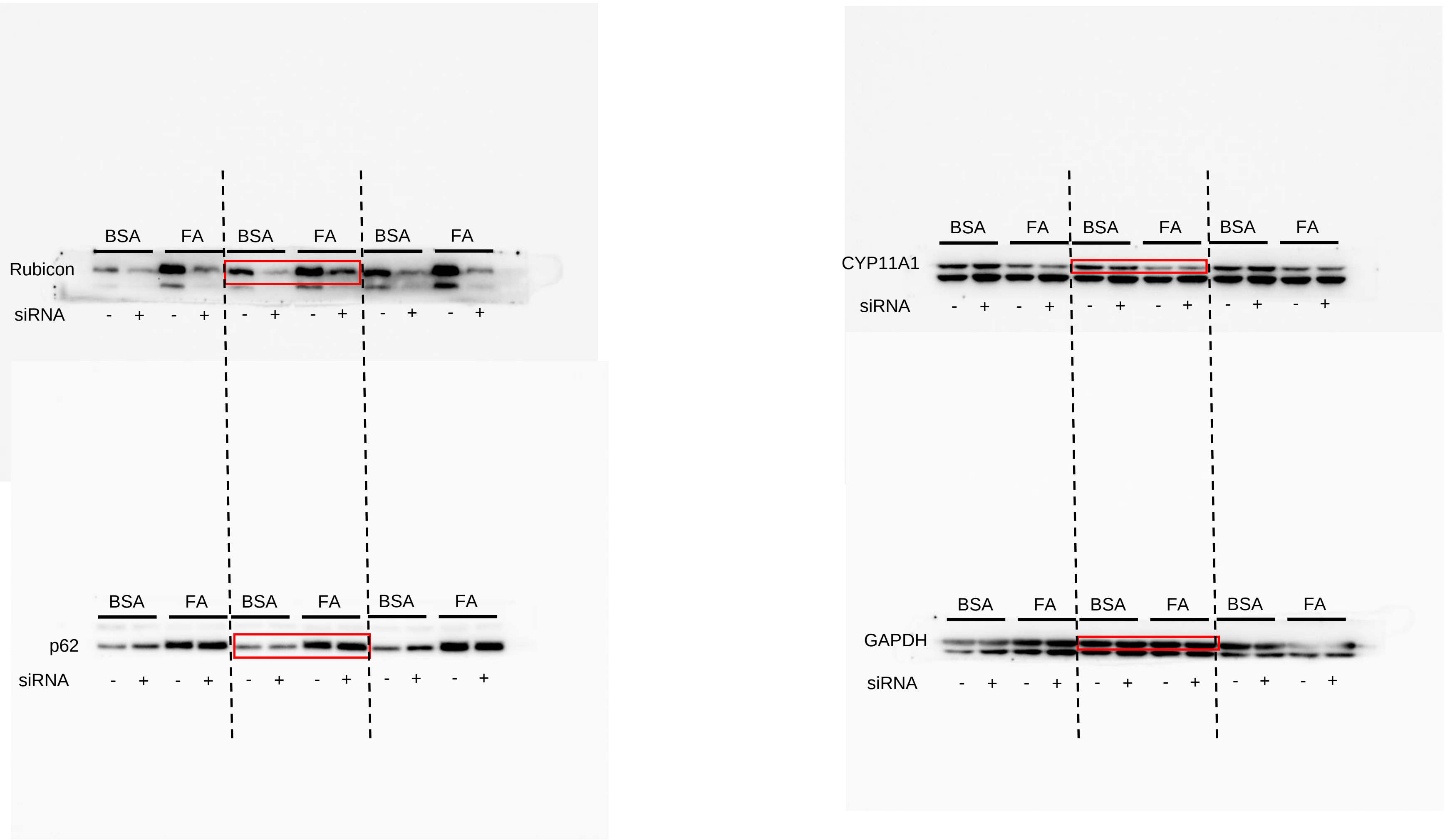

Supplementary Figure S6

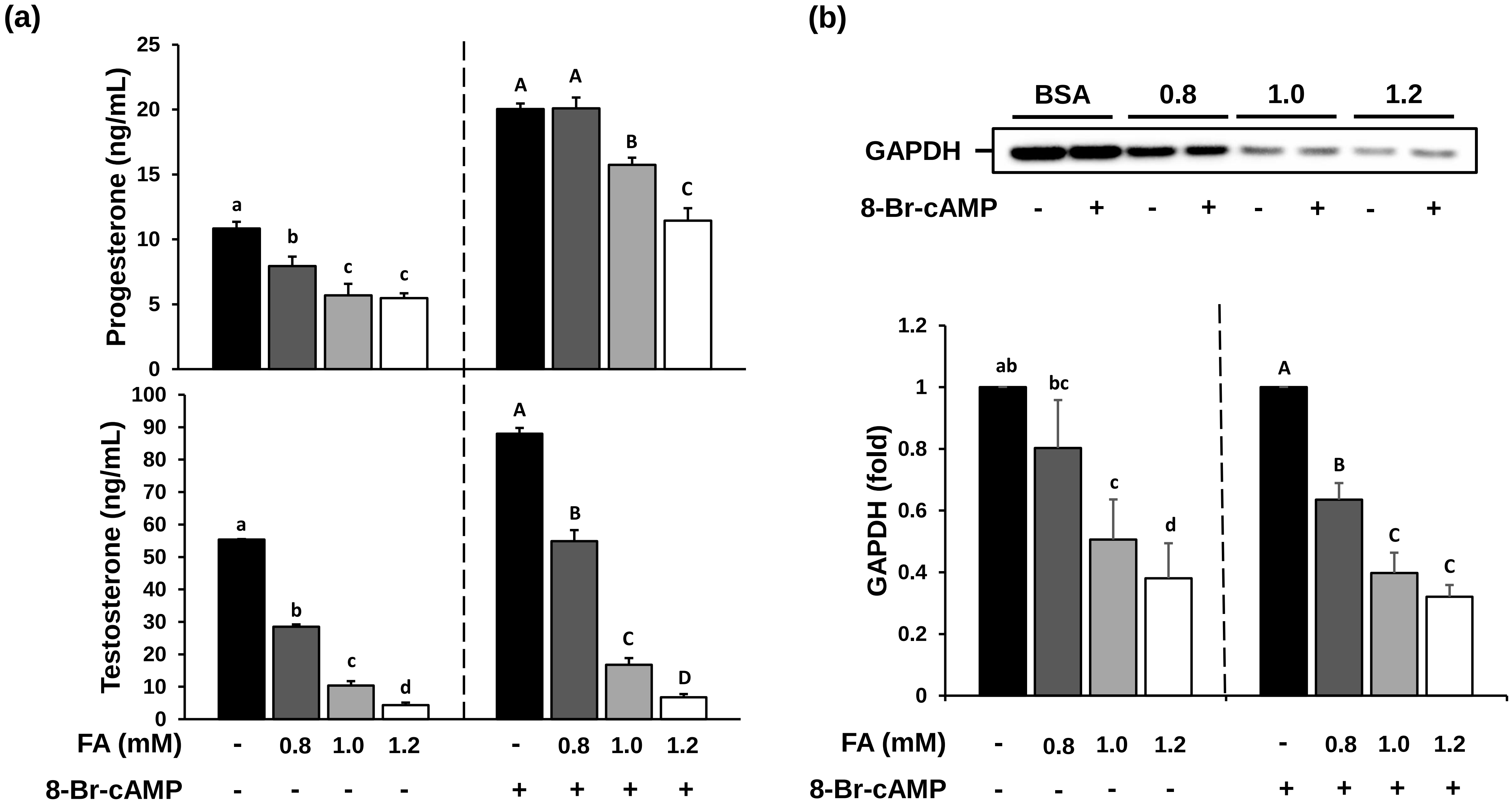

Supplementary Figure S7

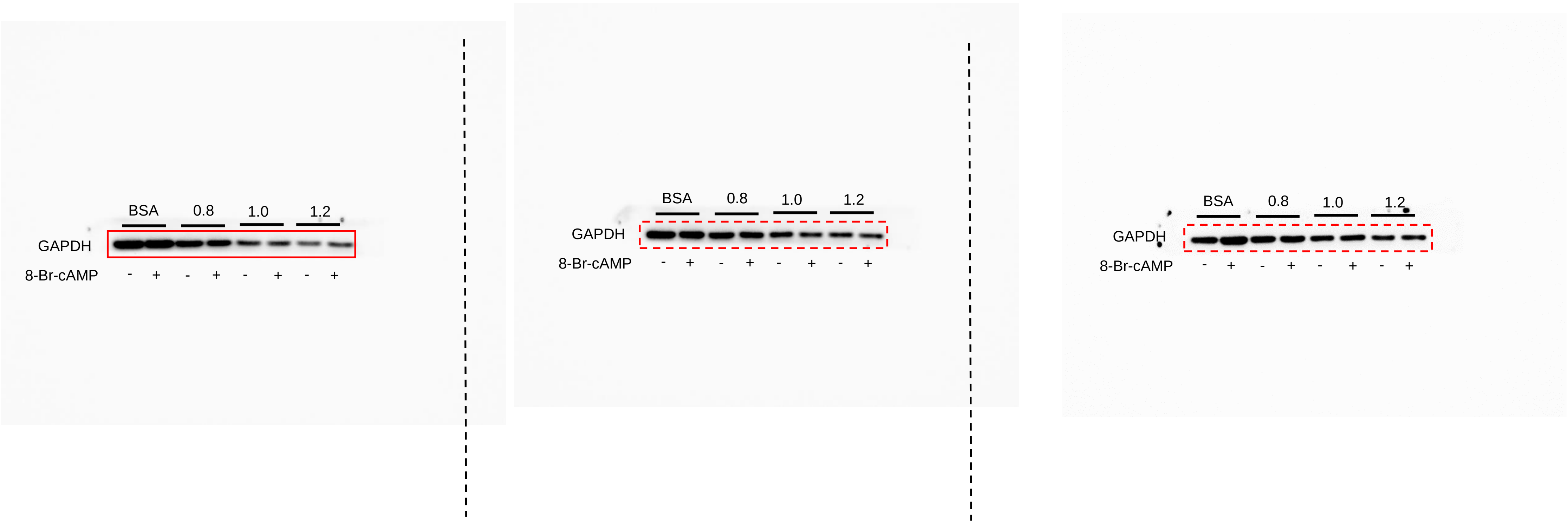

Supplementary Figure S8

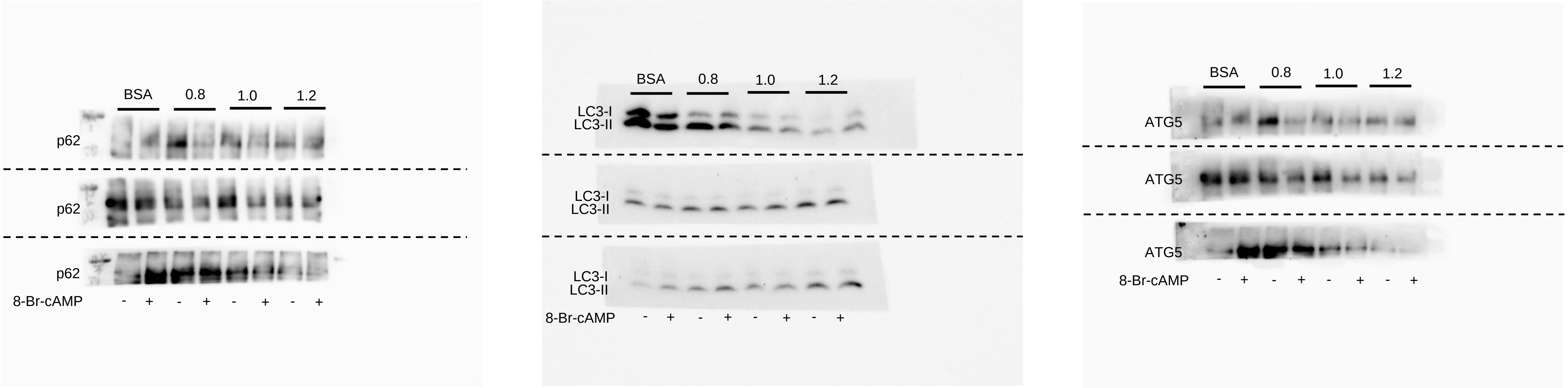

Supplementary Figure S9

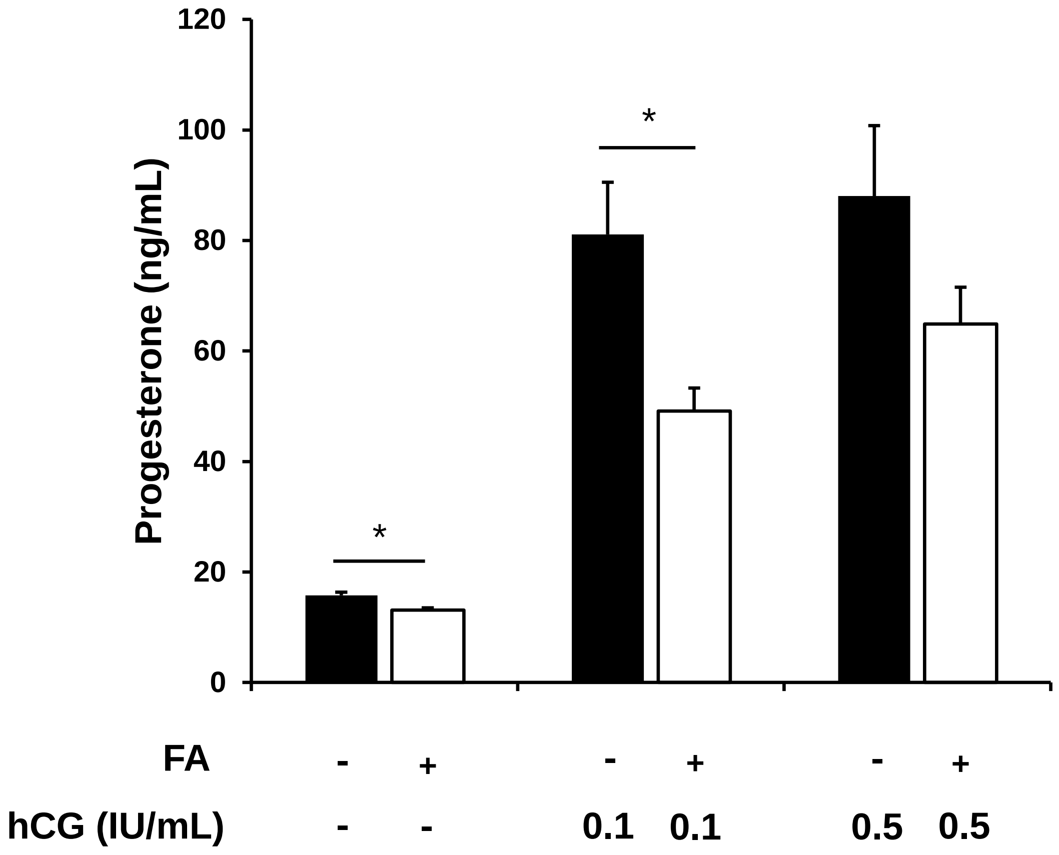

Supplementary Figure S10
